# Characteristics of Medical Students with Physician Relatives: A National Study

**DOI:** 10.15694/mep.2018.0000030.1

**Published:** 2018-02-02

**Authors:** Kwang Jin Choi, Hyo Jung Tak, Clark Bach, Cathleen Trias, Huma Warsi, Joseph Abraham, John D. Yoon

**Affiliations:** 1 The University of Chicago; 2 University of Nebraska Medical Center; 3 Mercy Hospital & Medical Center

**Keywords:** underserved, primary care, national survey, physician relative

## Abstract

This article was migrated. The article was marked as recommended.

Background

Little is known regarding U.S. medical students with close family relatives who are physicians. Family-related factors may influence students’ specialty decisions to enter primary care or practice among the underserved.

Methods

Self-administered questionnaires were sent to 960 third-year U.S. medical students from 24 U.S. allopathic medical schools in January 2011. We asked respondents whether or not they had a physician parent or grandparent. We also tested associations between physician relative status and demographics, educational factors and career intentions.

Results

Response rate was 61% (564/919). Among the respondents, 124 students (22.0%) responded that they had a physician relative. Students having a physician relative were less likely to intend to practice among the underserved and enter into a primary care specialty (all P-values < 0.05). Having a physician relative was negatively associated with intention to practice among the underserved (odds ratio, 0.37; 95% confidence interval, 0.16-0.87) in multivariate logistic estimation controlling for socio-demographics and educational factors.

Conclusion

Students who reported having a physician parent or grandparent were less likely to be reporting intentions to practice among the underserved. More studies are needed to identify whether physician relative status represents an important factor for future career trajectories or serve merely as a surrogate for other socioeconomic factors.

## Introduction

Despite efforts to address the growing need of primary care for the medically underserved, physician shortages persist in many areas of the U.S. ( Association of American Medical Colleges, 2016; Health Resources & Services Administration, 2016). Studies have identified predictors for working with the underserved including: being an underrepresented minority, growing up in an underserved area, identifying with medicine as a calling, and belonging to a family that emphasizes service to the poor ( Curlin, Dugdale, Lantos, & Chin, 2007; Goodfellow et al., 2016; Komaromy et al., 1996; Rabinowitz, Diamond, Veloski, & Gayle, 2000). Among various factors, family-related influences in medicine remain relatively unexamined. For example, one study found that physicians with highly educated spouses were less likely to work in rural underserved areas ( Staiger, Marshall, Goodman, Auerbach, & Buerhaus, 2016). In addition, medical students with close family members who are physicians may be influenced by shared familial commitments to care for the underserved ( Curlin et al., 2007). But very little is known in general about U.S. medical students with close family relatives who are physicians. Therefore, this study conducted a secondary data analysis of a national survey of third year U.S. medical students to describe the characteristics of those students with physician relatives. Specifically, we examined whether intentions to work for the medically underserved or go into primary care were associated with having a parent or grandparent who is a physician.

## Methods

The *Professional Development of Physicians-in-Training* project mailed a confidential, self-administered questionnaire to 960 third-year U.S. medical students in January 2011. The study sample was drawn from the American Medical Association Physician Master File (AMA-PMF), which includes nearly all medical students pursuing MD and DO degrees in the US and its territories. We designed a systematic survey sampling plan with probability proportional to size and implicit stratification to acquire a nationally representative individual-level data. With this survey design, we selected 24 U.S. allopathic medical schools, and then randomly sampled 40 students from each school. Further details of our methodology have been described elsewhere ( O’Connell, Ham, Hart, Curlin, & Yoon, 2018). The study was approved by the University of Chicago Social Sciences Institutional Review Board.

The outcome of interest are two binary variables of career intentions, indicating whether or not the respondent has an intention to practice among the underserved and enter into a primary care specialty. Primary explanatory variable is having a physician parent or grandparent. Other descriptive variables included socio-demographics (gender, region, underrepresented minority status, having grown up in a medically underserved setting) and educational factors (undergraduate major, educational debt by graduation, social mission score of medical school).

We used descriptive statistics to summarize student socio-demographics, educational factors, and career intentions by physician relative status. Bivariate analyses were performed with Pearson Chi-squared tests to investigate systematic differences by physician relative status. Multivariate logistic regression models were used to examine the associations of physician relative status and career intentions, after controlling for socio-demographics and educational factors. In order to make nationally representative estimates, case weights were constructed to reflect sources of variance associated with the sample design and to adjust for potential nonresponse bias. Analyses were performed using survey analysis procedures (i.e., probability weight, primary sampling units, and strata) in Stata MP v 14.2.

## Results

After excluding students who were not third year medical students, adjusted response rate was 61% (564/919). Among 564 total respondents, 124 students (22.0%) responded that they had a physician relative. Table 1 shows the descriptive statistics of various characteristics of medical students by physician relative status. When we examined socio-demographics, we found that students who have a physician relative were less likely to be underrepresented minorities and grow up in medically underserved settings (all P-values < 0.01). Gender and region were not statistically different by physician relative status. When we examined educational factors, students who have a physician relative were more likely to have a non-science/engineering undergraduate majors, have a lower educational debt, and come from lower ranked social mission medical schools (all P-values < 0.05). Lastly, when we examined career intentions, students who have a physician relative were less likely to intend to practice among the underserved and enter into a primary care specialty (all P-values < 0.05).

**Table 1.** Characteristics of medical students by physician relative status

	Physician relative?		P-value
	No (n = 440)	Yes (n = 124)	
	N (%)	N (%)	
**Demographics**			
Female	209 (47.4)	50 (40.2)	0.06
Region			0.12
Northeast	103 (23.3)	37 (29.9)	-
South	171 (39.1)	39 (31.6)	-
Midwest	108 (24.5)	32 (25.7)	-
West	58 (13.1)	16 (12.8)	-
Underrepresented minority	73 (16.5)	8 (6.2)	**< 0.01**
Grown up in a medically underserved setting	131 (29.8)	11 (8.6)	**< 0.01**
**Educational Factors**			
Science or engineering major in undergraduate			**0.03**
No	79 (17.9)	34 (27.4)	
Yes	361 (82.1)	90 (72.6)	
Expected total educational debt by graduation			**0.02**
$100,000 or less	252 (57.3)	88 (71.0)	-
$100,001 or above	188 (42.7)	36 (29.0)	-
Medical school ranking according to social mission score			**< 0.01**
Ranking: 1-30	117 (26.6)	21 (16.6)	
Ranking: 30-64	114 (25.9)	29 (23.6)	
Ranking: 65-110	103 (23.5)	37 (30.1)	
Ranking: 111-140	106 (24.0)	37 (29.7)	
**Career Intentions**			
Intentions for future specialty			**0.04**
Primary care	157 (35.7)	35 (28.0)	-
Specialist	283 (64.3)	89 (72.0)	-
Intentions to practice in a medically underserved setting	156 (35.4)	17 (13.7)	**< 0.01**

In multivariate logistic estimations, having a physician relative was negatively associated with intention to practice among the underserved (odds ratio [OR], 0.37; 95% confidence interval [CI], 0.16-0.87), after controlling for socio-demographics and educational factors. However, having a physician relative did not have a statistically significant association with an intention to enter into a primary care specialty.

## Discussion

In this secondary data analysis of a 2011 national survey of U.S. medical students, we found that medical students with intentions to practice among the underserved were less likely to report having a parent or grandparent who was a physician. This finding suggests a relatively unexamined factor in admissions to medical schools and residency training programs when selecting for those students who are likely to practice in medically underserved settings: whether or not a candidate has a physician relative.

Schwartz and his colleagues ( Schwartz, Linzer, Babbott, Divine, & Broadhead, 1991) found that the five most influential factors in medical students choosing general internal medicine were intellectual stimulation, continuity of care, the chance to contribute (pride in profession), coordinating patients’ care, and the academic challenge on rounds. In contrast, the five most influential factors pushing medical students away from general internal medicine were chronically ill patients, the type of patients seen in general internal medicine, the level of satisfaction, treating alcohol or drug abusing patients, and the degree of stress. Of the 1244 respondents in Schwartz’s article, 19% had a physician parent, but associations with this physician relative status and other variables were not examined in the study ( Schwartz et al., 1991). Another study by Henning and colleagues ( Henning, 2015) asked 193 physicians, “What do practicing physicians identify as the most significant influences on the selection of their area of specialty?” The majority of the respondents (nearly 60%) reported they had no family members who were physicians. Ten (5.0%) responded or chose “Influence of Physician Relatives” as an important factor in choosing their specialty. Nevertheless, little is known whether physician relatives may be influencing medical students away from certain medical careers, or whether medical students may be influenced by witnessing the personal challenges inherent in certain careers of their physician relatives, such as practicing among the underserved.

In our study, we found medical students with non-physician relatives were more likely to choose primary care careers than students with physician relatives, though this finding did not remain significant after controlling for various factors in our multivariate models. Currently, there are 778,000 practicing physicians in the United States. Approximately one half of them are engaged in primary care, but approximately one half are over the age of 50, and almost one third are projected to retire in the next 10 years. Compounding the problem is that currently, just 25% of medical school graduates go into and remain in primary care ( Morelli, 2017). Workforce analysis authors predict a shortage of physicians ranging from 12,500 to 31,000 by 2025 ( Association of American Medical Colleges, 2016), and this workforce shortage may be further compounded by the added difficulty of recruiting physicians to medically underserved areas.

This study has important limitations. First, this was a cross-sectional study so we could not assess causation between the various factors. Second, nonresponse bias may be affected the results of our study. Lastly, our analyses used data collected in 2011 so these associations would need to be further tested in future surveys to confirm these findings with contemporary trends in medical education.

Note:

i) Percentages were adjusted for probability weight, primary sampling unit, and strata in survey data analysis.

ii) P-values were calculated by Chi-squared test.

## Take Home Messages

• Data from a 2011 national survey of third year medical students found that those students who reported having a physician parent or grandparent were less likely to be reporting intentions to practice among the underserved.
• More studies are needed to identify whether physician relative status represents an important factor for future career trajectories, or whether this status represents a surrogate marker for other socioeconomic factors.

## Notes On Contributors

Kwang Jin Choi, MD is Clinical Associate in the Department of Medicine at the University of Chicago.

Hyo Jung Tak, PhD is Assistant Professor in the Department of Health Services Research and Administration at the University of Nebraska Medical Center.

Clark Bach, MD is PGY-3 Resident in Internal Medicine at Mercy Hospital & Medical Center.

Cathleen Trias, MD is PGY-3 Resident in Internal Medicine at Mercy Hospital & Medical Center.

Huma Warsi, MD is PGY-3 Resident in Internal Medicine at Mercy Hospital & Medical Center.

Joseph Abraham, MD is Program Director of the Internal Medicine Residency Program at Mercy Hospital & Medical Center.

John D. Yoon, MD is Assistant Professor in the Department of Medicine at the University of Chicago.
