# Supplementary material for: Characteristics of Medical Students with Physician Relatives: A National Study
Source: MedEdPublish (2016). 2018 Feb 2;7:30. [Version 1] doi: 10.15694/mep.2018.0000030.1 (PMC10712000; doi:10.15694/mep.2018.0000030.1)
Supplement: Peer review history

# Reviewer report: Reviewer response for version 1

**DOI:** 10.21956/mep.19501.r28827

**Published:** 2018-03-10

**Authors:** Mohamed Al-Eraky

**Affiliations:** 1 University of Dammam

This review has been migrated. The reviewer awarded 3 stars out of 5 That's an interesting study. I Agree with Subha that the conclusion might be misleading as medical schools may give priority to applicants who are sons and daughters of physicians. Then they may even go further to filter who among those physician parents were successful or not. I still find a high potential of the study, particularly when a couple of clear research questions are indicated from start to expand the scope of the study. Since they may have access to students' grades and demographics, authors can make many possible correlations and may come up with conclusions like those presented in the book of Steve Levitt: Freakonomics. Thank you.

Reviewer Expertise:

NA

No decision status is available

# Reviewer report: Reviewer response for version 1

**DOI:** 10.21956/mep.19501.r28825

**Published:** 2018-02-05

**Authors:** Maurizio Vertemati

**Affiliations:** 1 Dipartimento di Scienze Biomediche e Cliniche "L. Sacco"

This review has been migrated. The reviewer awarded 2 stars out of 5 In this interesting study, the main conclusion drawn by the authors was that physician relative status could be involved in future career trajectories.That observation is supported by their data, but it could be interesting to investigate what is the progress of these students according to their physician relative status. Moreover, another point to analyze could be the impact of the new strategies in medical education on the choice of future carreer.I think that this work is an interesting work-in-progress study which represents a firts step to evaluate the role of physician relative status in medical students and that needs to be worth considering.

Reviewer Expertise:

NA

No decision status is available

# Reviewer report: Reviewer response for version 1

**DOI:** 10.21956/mep.19501.r28828

**Published:** 2018-02-03

**Authors:** Trevor Gibbs

**Affiliations:** 1 AMEE

This review has been migrated. The reviewer awarded 2 stars out of 5 I always find it quite difficult to positively review a paper when it feels that the research is only half way though. I was left wanting with this paper, which I suppose could count as an initial inquiry.Given its implications I hope that the authors can continue with a more expansive piece of research that would look through a more qualitative lens at this possible issue- it would certainly make for interesting debate as to who and how we choose our students ready for the real 21st century.

Reviewer Expertise:

NA

No decision status is available

# Reviewer report: Reviewer response for version 1

**DOI:** 10.21956/mep.19501.r28826

**Published:** 2018-02-02

**Authors:** Subha Ramani

**Affiliations:** 1 Harvard Medical School, Brigham and Women's Hospital

This review has been migrated. The reviewer awarded 4 stars out of 5 This is an interesting study carried out by the investigators. Practicing in underserved communities and taking on primary care roles are important outcomes with reported shortage of primary care physicians. It certainly would be important for medical schools which emphasize careers in primary care to ascertain what factors would facilitate or impede students seeking primary care career or working in underserved communities.The study is well written and the authors make a case for why they did this study. They raise the possibility that having physician family members could play in future professional trajectories.However, as they themselves state, this was a cross sectional survey with no exploration of the reasons for the findings. I acknowledge that a qualitative approach would not be possible with the distribution and numbers of students. But, exploring opinions of multiple groups of students in different geographical distributions would be important. Otherwise, schools might start looking at physician families as detriments on selection criteria.This is a provocative query, but more details are necessary before conclusions can be made about career interests.Educational leaders working on admissions committees at medical schools and faculty who mentor and provide career advice to students as well as primary care leadership will find these results intriguing.

Reviewer Expertise:

NA

No decision status is available

# Reviewer report: Reviewer response for version 1

**DOI:** 10.21956/mep.19501.r28824

**Published:** 2018-02-02

**Authors:** P Ravi Shankar

**Affiliations:** 1 American International Medical University

This review has been migrated. The reviewer awarded 4 stars out of 5 I enjoyed reading this manuscript on the characteristics of medical students who have physician relatives. The practice preferences and specialty choices of medical students is an area of interest to healthcare planners. Many countries face a shortage of primary care physicians and many physicians are reluctant to serve in underprivileged and under-served areas. Larger studies on factors determining the practice locations and specialty choices of medical graduates are required. The authors have mentioned physician relatives but I believe in the Methods section they have referred to this as having a physician parent or grandparent. In some cases, physician relatives other than parents could also influence the specialty choice and practice locations of students. As mentioned by Dr Ramani if confirmed by larger studies the results could have implications for medical school admission committees.

Reviewer Expertise:

NA

No decision status is available
